# A Novel Follitropin Analog Inhibits Follitropin Activity In Vitro

**DOI:** 10.3390/pharmaceutics13030325

**Published:** 2021-03-03

**Authors:** Naiel Azzam, Rinat Bar-Shalom, Fuad Fares

**Affiliations:** 1 MIGAL Galilee Research Institute, Kiryat Shmona 11016, Israel; naiela@migal.org.il; 2 Department of Human Biology, Faculty of Natural Sciences, University of Haifa, Haifa 3498838, Israel; rbar-shal@univ.haifa.ac.il

**Keywords:** FSH, FSH receptor, glycoprotein hormones, *N*-linked oligosaccharides, antagonist

## Abstract

Follitropin (FSH) is a heterodimeric protein composed of an *α* subunit that is shared with the glycoprotein hormone family, including lutropin (LH), thyrotropin (TSH), human choriogonadotropin (hCG), and a unique *β* specific subunit. Both *α* and FSH*β* subunits contain two sites of *N*-linked oligosaccharides, which are important for its function. FSH has a crucial function in the reproductive process in mammals. However, there are some clinical conditions, such as menopausal osteoporosis or adiposity, associated with increased FSH activity. Moreover, in some cases, carcinogenesis is evidently associated with activation of FSH receptor. Therefore, developing a follitropin antagonist might be beneficial in the treatment of these conditions. Here, we describe a novel, engineered, non-glycosylated single-chain FSH variant, prepared by site-directed mutagenesis and fusion of the coding genes of the *α* and *β* subunits. The designed variant was expressed in Chinese hamster ovary (CHO) cells and successfully secreted into the culture medium. We found that the non-glycosylated single-chain FSH analog binds with high affinity to FSH receptor and efficiently inhibits FSH activity in vitro. This variant acts at the receptor level and has the potential to serve as a follitropin antagonist for clinical applications in the future.

## 1. Introduction

Follitropin (FSH) is a member of the glycoprotein hormone family, which includes lutropin (LH), thyrotropin (TSH) and human choriogonadotropin (hCG). These hormones are heterodimeric proteins that share a common *α* subunit, but differ in their unique *β* subunit, which confers biological specificity [1]. The specific *β* subunit of each hormone non-covalently associates with the *α* subunit to form the active hormone. The assembly of the subunits is critical for the secretion and activity of the hormones. Glycoprotein hormones activate the target cells via their specific G protein-coupled receptors (GPCRs), expressed on cell membranes [2]. In the case of FSH, the activation of the receptor leads to increased steroid synthesis [3] and production of reproductive hormones [4].

In males, FSH induces proliferation of Sertoli cells in the testis and initiates and maintains normal spermatogenesis [5]. In females, FSH initiates follicular growth in the ovary and induces maturation of the follicle [3], specifically affecting granulosa cells of the follicle to secrete estrogens [6]. Nevertheless, some clinical conditions have been associated with increased FSH levels and FSH receptor (FSHR) activity, as result of ovarian failure in the menopausal period. These conditions include enhanced osteoporosis, obesity [7] and carcinogenesis [8]. Therefore, developing an antagonist for FSH at the receptor level might be useful in treatment of such conditions.

As a glycoprotein hormone, the FSH*β* subunit carries two asparagine (Asn) *N*-linked oligosaccharide chains at positions Asn-7 and Asn-24. Additionally, the *α* subunit carries two *N*-linked oligosaccharide chains at positions Asn-52 and Asn-78 [2]. The oligosaccharides on the glycoprotein hormones have been implicated in several actions, including the maintenance of intracellular stability, secretion, assembly, receptor binding, steroidogenesis and modulation of plasma half-life [2]. It is known that non-glycosylated FSH subunits hardly assemble, and the fully non-glycosylated dimer is not secreted from the cells [9].

Therefore, by using site-directed mutagenesis and gene fusion, we designed and constructed a novel non-glycosylated single-chain FSH analog, to be evaluated as an antagonist of FSH. We hypothesized that the conversion of the dimeric FSH to single-chain construct will enable efficient secretion of this novel protein from transfected cells, despite being fully non-glycosylated. Moreover, this variant would inhibit FSH activity.

## 2. Results

### 2.1. Expression of Single-Chain FSH Variants

**Figure 1:**
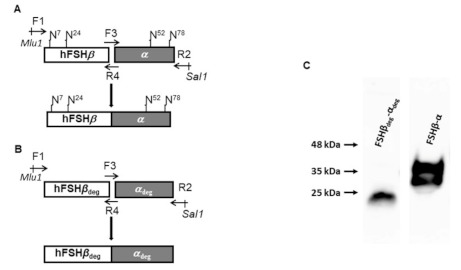
Construction of chimeric single-chain FSH variants using site-directed mutagenesis and overlapping PCR and expression in mammalian cells. The chimeric coding genes for fully glycosylated single-chain, FSH*β*-*α*, and fully non-glycosylated, FSH*β*_deg_-*α*_deg_, variants were constructed by fusing the respective *α* and *β* subunits, using overlapping PCR (**A**,**B**). The *β* subunits (FSH*β* and FSH*β*_deg_) were initially amplified with primers F1 and R4 and the *α* subunits (*α* and *α*_deg_) with primers F3 and R2. F3 and R4 are overlapping primers that code for the 3‘ end of FSH*β* and the 5‘ start of the *α* coding gene. The obtained fragments were used as a template for overlapping PCR with the edge primers, F1 and R2. Primers F1 and R2 respectively carry *Mlu*1 and *Sal*1 restriction sites, which served for cloning into the eukaryotic expression vector, pCI-DHFR. (**C**) Secreted single-chain FSH variants from transfected CHO/dhFr cells were detected by gel electrophoresis and Western blot, using an anti-FSH*β* antibody. The positions of the molecular mass markers are indicated in kDa.

CHO-dHFr^−^ cells were transformed with the chimeric genes (Figure 1A,B) coding for the FSH*β*-*α* and FSH*β*_deg_-*α*_deg_ single-chain variants. The expression and secretion of FSH variants to the media were confirmed by Western blot. Figure 1C illustrates the migration of the variants, which occurred according to the carbohydrate chain content. The non-glycosylated variant (FSH*β*_deg_-*α*_deg_) migrated faster than the fully glycosylated FSH*β*-*α*. The net molecular weight exhibited by FSH*β*_deg_-*α*_deg_ was ~24 kDa. The FSH*β*-*α* single chain appeared as a major band of molecular weight of ~38 kDa and a lower band with molecular weight of ~33 kDa.

### 2.2. Receptor Binding Assays

**Figure 2:**
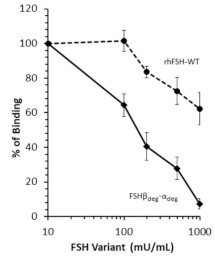
Evaluation of receptor binding affinity of FSH variants. CHO cells stably expressing the human FSH receptor were incubated with 100 mU/ mL of biotin-labeled FSH*β*-*α* single-chain protein with ascending concentrations of FSH variants (0, 100, 200, 500 and 1000 mU/mL). The results were calculated as the percentage of bound biotin-FSH*β*-*α* at each concentration of the FSH variant. The total binding was defined as the binding of labeled protein without variant. Data shown are the mean ± SE of 5 different experiments (*n* = 5) with 3 replicates for each concentration in the experiment. Statistical analysis was carried out using one-way ANOVA followed by Tukey HSD multiple comparison test. Two-way ANOVA was performed for comparison between FSH variants. Significance was found in both tests (*p* < 0.001).

The receptor binding assays were carried out in order to evaluate the potential of FSH*β*_deg_-*α*_deg_ to displace labeled single chain FSH*β*-*α* on the membrane follitropin receptor (FSHR), as compared to recombinant human FSH-WT (rhFSH-WT). The results are presented as the percentage of bound biotin-FSH*β*-*α* as a function of ascending concentrations of the respective unlabeled FSH variant (Figure 2). At 1000 mU/mL, the rhFSH-WT could displace only 38% of biotin-FSH*β*-*α*, while the FSH*β*_deg_-*α*_deg_ displaced ~93% of the biotinylated ligand. The difference between the displacement potentials of the FSH variants was statistically significant (*p* < 0.001).

### 2.3. Activity of FSH Variants

**Figure 3:**
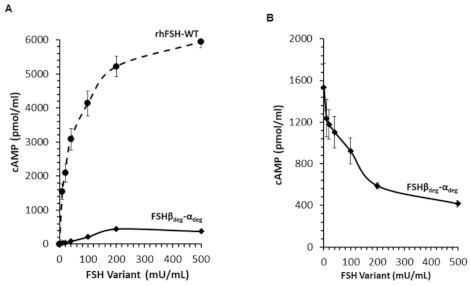
In vitro activity of FSH variants. (**A**) Biological activities of FSH variants were determined by measurement of induced cAMP in the media. CHO-dhFr cells stably expressing FSHR were exposed to ascending concentrations of FSH variants (0–500 mU/mL) for 20 h. Each curve is the mean ± SE of at least 5 independent experiments (*n* ≥ 5), with 3 replicates for each concentration in each experiment. One-way ANOVA showed a dose-dependent increase in activity following exposure to rhFSH-WT (*p* < 0.001). Two-way ANOVA indicated significant differences between FSH variants (*p* < 0.001). (**B**) Competitive activity of single-chain FSH*β*_deg_-*α*_deg_ vs rhFSH-WT. FSHR-expressing CHO cells were incubated for 20 h with 10 mU/mL of rhFSH-WT and with ascending concentrations of FSH*β*_deg_-*α*_deg_ (0–500 mU/mL). One-way ANOVA indicated significant effects at 200 and 500 mU/mL (*p* < 0.001).

The biological activity of the engineered FSH*β*_deg_-*α*_deg_ was determined and compared to that of rhFSH-WT. Following exposure of FSHR-expressing cells to increasing concentrations of FSH variants, the amounts of cAMP produced were measured. Despite the high binding affinity of FSH*β*_deg_-*α*_deg_ to FSH receptor, it demonstrated negligible activity (~2–10%) compared to that of rhFSH-WT (*p* < 0.001) (Figure 3A).

In order to evaluate the antagonistic potential of the FSH*β*_deg_-*α*_deg_ variant, competitive bioactivity experiments were carried out in the presence of rhFSH-WT (10 mU/mL). The FSH*β*_deg_-*α*_deg_, variant competed with rFSH-WT in a dose-dependent manner (Figure 3B). The activity of rhFSH-WT was gradually reduced by 62% and by 73% in the presence of 200 mU/mL and 500 mU/mL of FSH*β*_deg_-*α*_deg_, respectively (*p* < 0.001).

## 3. Discussion

In the present study, we designed and constructed a novel analog of FSH and evaluated its potential to inhibit FSH in vitro. *N*-linked glycosylation has been postulated to be involved in secretion and bioactivity of glycoproteins, including FSH [2]. Deglycosylation of glycoprotein hormones was shown to decrease bioactivity but not receptor binding affinity [2]. This suggested that non-glycosylated hormone analogs could competitively antagonize and inhibit the biological activities of the native hormones. Previously, deglycosylation of glycoproteins was carried out using chemical or enzymatic treatments that could affect the protein structure or even cause damage to its backbone [2]. Thus, removal of *N*-oligosaccharide chains by site-directed mutagenesis would be more promising. However, the attempts to express totally non-glycosylated dimeric FSH have not been successful in the sense that fully non-glycosylated subunits hardly assembled and therefore, the dimers were not secreted [9].

To overcome the limitation of subunit assembly, we designed a new, fully non-glycosylated FSH construct by fusing the mutated FSH subunits to form a single-chain variant. Indeed, it was indicated that our novel, non-glycosylated single-chain FSH variant, FSH*β*_deg_-*α*_deg_, was efficiently secreted from the cells to the culture medium, overcoming the critical step of subunit assembly.

Following gel electrophoresis and Western blotting, FSH*β*_deg_-*α*_deg_ appeared as one single band of ~24 kDa, representing more or less the sum of the net molecular weights of fully non-glycosylated FSH*β* and *α* subunits (14.7 kDa and 10.5 kDa, respectively). The FSH*β*-*α* single chain was detected in two-size forms, one of ~38 kDa and the other of ~33 kDa. The difference in the migration of the two forms of FSH*β*-*α* single chain may be attributed to macro-heterogeneity of patterns of glycosylation. This kind of heterogeneity was reported for hCG as well as for dimeric hFSH [10,11,12,13]. We assume, therefore, that the two-band appearance of the FSH*β*-*α* single chain in the current study is due to partial and complete glycosylation patterns.

Knowing that native FSH (as well as rhFSH-WT) and single-chain FSH*β*-*α* have similar affinities to the FSHR receptor [14], the receptor binding assays used in the present study were performed using labeled single-chain FSH*β*-*α* with biotin. The main advantage for the use of biotin-labeled single-chain FSH*β*-*α* is its higher stability, when compared to that of dimeric rFSH-WT; this due to the fact that *α* and *β* subunits do not dissociate. In this study, the FSH*β*_deg_-*α*_deg_ exhibited noticeably higher affinity to the FSHR compared to that of the glycosylated dimeric rhFSH-WT and almost fully displaced the biotin-labeled ligand.

In principle, hypo-glycosylation causes the FSH variants to be more compact and enables better binding with higher affinity to FSHR [15]. Hypoglycosylated dimeric FSH preparations at either *N*-linked site on the *β* subunit were found to occupy two-fold more binding sites on the receptor than the fully glycosylated FSH. The glycosylation rate of the hormone seems therefore to determine the number of the available receptors for hormone binding, due to allosteric considerations [16]. Since FSH receptors are known to form at least dimers, it was observed that binding of one molecule of FSH allosterically causes the dissociation of the other molecule of FSH that was previously bound to the FSHR dimer [16]. Therefore, the greater binding activity of the fully non-glycosylated FSH*β*_deg_-*α*_deg_ to the FSH receptor is well understood.

Despite the high binding affinity of FSH*β*_deg_-*α*_deg_ to FSH receptor, it demonstrated negligible activity when comparing to that of rhFSH-WT. This reconfirms the role of *N*-linked carbohydrates in FSH activity. The competitive experiments between FSH*β*_deg_-*α*_deg_ and rhFSH-WT revealed a dose-dependent inhibition of FSH activity by the non-glycosylated variant. This result illustrates the potential of FSH*β*_deg_-*α*_deg_ to be further evaluated as an antagonist of FSH.

In accordance with our findings, Flack et al. showed that hetero-dimeric hFSH composed of either non-glycosylated FSH*β* (FSH*β*_deg_/*α*) or non-glycosylated *α* (FSH*β*/*α*_deg_) exhibited higher receptor binding affinity, but lower bioactivity, than the fully glycosylated wild-type hormone in rat granulosa cells [17]. Other studies seem to contradict our study in the sense that they reported similar or increased activity of hypo-glycosylated FSH glyco-forms, compared to that of FSH-WT [18,19,20]. However, it should be emphasized that all these studies were conducted with dimeric molecules of FSH glyco-forms. The presence or absence of any of the *N*-linked oligosaccharide chains would differentially affect the receptor binding and the signal transduction. Jiang and colleagues gained evidence that the FSHR exists in functional trimers. They proposed a model according to which only one molecule of fully glycosylated FSH dimer might bind to the trimer of FSHR. On the contrary, this trimer could bind three molecules of FSH, non-glycosylated at position Asn52 of the *α* subunit [21]. An additional model explaining the linkage between receptor binding affinity and FSH activity was further proposed by Natajara and colleagues. They suggested that the basal state of FSHR is an inactive trimer. However, when a fully glycosylated FSH binds to the receptor, the trimer dissociates and the monomeric FSH-FSHR complex becomes active. In contrary, binding of three molecules of non-glycosylated FSH stabilizes the FSHR trimer and keeps it inactive [22].

Relying on all the above, we assume that engineered FSH*β*_deg_-*α*_deg_ exhibits high affinity to the FSH receptor because it is fully deprived of oligosaccharides. The complete deglycosylation confers a compact structure, enabling rapid and favorable binding, but significantly diminishes the activity.

FSH elevation as a result of ovarian failure has been implemented in osteoporosis and obesity in menopause [7,23] as well as in postmenopausal atherosclerosis [24]. In addition, numerous works have proposed a role for FSH and its receptor in carcinogenesis, mostly in ovarian cancer [25,26,27], but also in a wide range of cancers. FSHR receptor expression has been detected in the blood vessels of many cancers, suggesting a role for FSH and FSHR in angiogenesis, via induction of vascular endothelial growth factor (VEGF) (8). Thus, the development of an FSH antagonist will be of enormous benefit in the treatment of clinical conditions associated with elevated FSH and FSHR activities.

Further studies are needed to explore the interaction of the novel, non-glycosylated single-chain variant with its FSH GPCR and to evaluate its potential to antagonize FSH activity in vivo. We are aware that non-glycosylated glycoproteins might be rapidly cleared from the circulation [28]. Therefore, we assume that strategy of developing single-chain FSH variants will be advantageous since it is assumed to confer high stability and elongated half-life in the circulation. Additionally, we are aware of the need to evaluate the immunogenicity of our engineered variants in future in vivo studies. We believe that our current findings set the basis for future studies that may lead to the development of FSH antagonists to be used in the clinic.

## 4. Materials and Methods

### 4.1. Construction of hFSH Variants

The genes including the exons and introns of the glycosylated and non-glycosylated *α* and FSH*β* subunits were kindly supplied by Prof. Irving Boime (Washington University, Saint-Louis, MO, USA). The codons of Asn at the glycosylation recognition sites (Asn-X-Ser/Thr) of *α* and *β* subunits were converted to the codons of aspartic acid and glutamine, respectively, by site-directed mutagenesis as described before [29,30]. Using overlapping PCR and specific primers (Table 1), the FSH*β* and *α* genes were fused to form fully glycosylated single-chain FSH (FSH*β*-*α*) (Figure 1A) and non-glycosylated single-chain FSH (FSH*β*_deg-_*α*_deg_) (Figure 1B).

### 4.2. Cloning of FSH Single-Chain Variants

The constructed chimeric genes coding for the FSH single-chain variants were cloned into the pCI-DHFR vector via *Mlu*1 and *Sal*1 sites. Direct Sanger sequencing was used to verify the sequences of the coding genes of the single-chain FSH variants including the desired mutations.

### 4.3. DNA Transfection and Clone Selection

Chinese hamster ovary cells, which are DHFR negative (CHO/dhFr^-^, ATCC^®^ CRL-9096™), were transfected with the plasmids using Mirus transfection reagent (Mirus Bio LLC, Madison, WI, USA) and grown for three weeks in selective CD DG44 ISCOVES medium without hypoxanthine and thymidine, supplemented with 10% dialyzed fetal calf serum. Only successfully transfected cells that received the DHFR gene were able to survive.

In order to carry out receptor binding and bioactivity assays, CHO/dhFr^-^ cells were stably transfected with the cDNA of human FSH receptor (FSHR) (ORIGINE, Rockville, MD, USA). The FSHR coding gene was cloned into the pCI-DHFR expression vector via *EcoR1* and *Sma1* restriction sites. Surviving cell clones were tested for existence of active FSHR, by evaluating their potential to produce cAMP following exposure to 10 mU of dimeric commercial recombinant human FSH-WT (rhFSH-WT). Samples of media from cell clones were taken for cAMP measurements using a Parameter^TM^ cAMP Assay kit according to the manufacturer’s manual. The cell clone harboring the most active FSHR was chosen for the receptor binding and biological activity assays.

### 4.4. Expression of FSH Variants

Media were collected from stable clones. Samples were electrophoresed on denaturing 10% sodium dodecyl sulfate-polyacrylamide gels and blotted to a nitrocellulose membrane. Following blocking with 5% nonfat dry milk, the membrane was incubated overnight with FSH*β* antibody (#ab-171431, abcam, Cambridge, UK). The next day the membrane was exposed to secondary antibody conjugated to horseradish peroxidase, and reacted with enhanced chemiluminescent substrate. An Amersham imager 600 gel scanner served for detection of protein bands on membranes.

Media from clones secreting FSH variants were collected and concentrated using a VIVAFLOW concentrating system, consisting of a peristaltic pump and a cut-off membrane of 10,000 Daltons. The media samples were further concentrated through VIVASPIN 20 concentrating devices with a cut-off of 10,000 Da (Sartorious Stedim Biotech, Goettingen, Germany) by centrifugation at 4000× *g*. The concentrations of FSH variants were determined using FSH ELIZA kit (ENZO Life Sciences (Farmingdale, NY, USA). These were found to be 2.7 U/mL and 200 U/mL for FSH*β*_deg-_*α*_deg_ and FSH*β*_-_*α*, respectively.

### 4.5. Receptor Binding Assays

Receptor binding assays of non-glycosylated single-chain FSH*β*_deg-_*α*_deg_, compared to dimeric FSH, were performed using biotin-labeled single-chain recombinant FSH*β-α* protein. For stability considerations, the FSH*β-α* variant was labeled in our laboratory with biotin using Ez-link Micro-Sulfo-NHS-Biotinylation kit (ThermoScientific, Rockford, IL, USA). CHO/dhFr cells which were stably transfected with the human FSHR gene and permanently expressed FSHR, were seeded in 24-well plates (2 × 10^5^ cells/well). On the following day, cells were washed and fixed with cold methanol for 10 min at −20 °C. Then, cells were exposed for 2 h to increasing concentrations of FSH variants (FSH*β*_deg-_*α*_deg_, and hFSH-WT) vs fixed concentration of 100 mU/mL of biotin-labeled single-chain FSH*β-α* protein. Commercial human recombinant FSH-WT (bioWORld, Dublin, OH, USA) was used for comparison. Thereafter, cells were washed and incubated with streptavidin-HRP (R&D Systems Inc., Minneapolis, MN, USA) in the dark for 1 h. Finally, tetramethylbenzidine (TMB) (Scy Tek, Logan, UT, USA) was added to the cells for 30 min in dark. The reaction was stopped by addition of 2N HCl stop solution. Plates were read in an ELIZA reader at 450 nm with correction at 540 nm.

### 4.6. In Vitro Activity

CHO/dhFr cells stably expressing FSHR were stimulated with increasing amounts of FSH variants for 20 h. The cAMP concentrations in media samples were determined using Parameter^TM^ cAMP Assay (R&D Systems Inc., Minneapolis, MN, USA) according to the manufacturer’s protocol.

### 4.7. Competitive Bioactivity Studies

CHO/dhFr cells expressing FSHR (2 × 10^5^ cells/well plated in 24-well plates) were exposed to increasing concentrations of FSH variant (0–500 mU/mL) in the presence of a fixed amount (10 mU/mL) of rhFSH-WT for 20 h. Media samples were collected and analyzed for the amounts of induced cAMP, as described above.

### 4.8. Statistical Analysis

Each experiment was repeated at least 5 times. The results were expressed as the mean ± standard error (SE). Statistical analysis of data was performed using one-way ANOVA followed by Tukey HSD multiple comparison and/or two-way ANOVA for comparison between FSH variants. All assays were performed with the SPSS-21 software (IBM spss statistics 21, 2020), and *p* < 0.05 was considered statistically significant.

## Figures and Tables

**Table 1:** The primers used for gene amplification in PCR reactions.

F1.	5′-AATGATACGCGTTCTTTGGTTTCTCAGTTT-3′
F3.	5′-GGTGAAATGAAAGAAGCTCCTGATGTGCAG-3′
R4.	5′-CTGCACATCAGGAGCTTCTTTCATTTCACC-3′
R2.	5′-CGGCGCGTCGACTTAAAAGGCTTTATTTGC-3′
FSHR-forward.	5′-TTTTTTGAATTCATGGCCCTGCTCCTGGTC-3′
FSHR-reverse.	5′-TTTTTTCCCGGGTTAGTTTTGGGCTAAATG-3′

## Data Availability

Data is available upon request from the corresponding author.
